# Supplementary material for: Effectiveness and Safety of Ayurvedic Medicines in Type 2 Diabetes Mellitus Management: A Systematic Review and Meta-Analysis
Source: Front Pharmacol. 2022 Jun 8;13:821810. doi: 10.3389/fphar.2022.821810 (PMC9213670; doi:10.3389/fphar.2022.821810)
Supplement: Supplementary file 1 [file DataSheet1.zip › Supplementary Material/Forest and Funnel Plots/Zingiber officinale/SBP.pdf]

Zingiber officinale - SBP

Study	Intervention N	Intervention Mean	Intervention SD	Comparator N	Comparator Mean	Comparator SD	Mean Diff. with 95% CI	Weight (%)
Arablou 2014a, Arablou 2014b	33	123.90	15.47	30	122.10	15.47	1.80 [-5.85, 9.45]	17.09
Azimi 2014, Azimi 2016	41	142.07	1.28	39	137.20	1.25	4.87 [4.32, 5.42]	26.61
Mozaffari-Khosravi 2014, Talaei 2017, Talaei 2018	40	109.00	11.27	41	117.31	12.70	-8.31 [-13.54, -3.08]	21.13
Arzati 2017, Zarezadeh 2018	23	120.00	14.70	22	117.00	13.80	3.00 [-5.34, 11.34]	16.00
Carvalho 2020	47	130.77	18.69	56	125.32	14.36	5.45 [-0.94, 11.84]	19.17
**Overall**							1.37 [-3.90, 6.64]	

Heterogeneity: $\tau^2 = 27.10$, $I^2 = 82.55\%$, $H^2 = 5.73$

Test of $\theta_i = \theta_j$: $Q(4) = 24.86$, $p = 0.00$

Test of $\theta = 0$: $z = 0.51$, $p = 0.61$

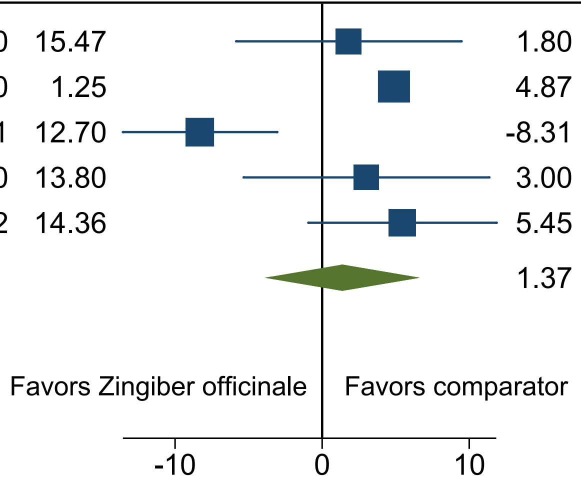

Random-effects REML model
